# The plant pathogen *Gluconobacter cerinus* strain CDF1 is beneficial to the fruit fly *Bactrocera dorsalis*

**DOI:** 10.1186/s13568-017-0514-y

**Published:** 2017-11-17

**Authors:** Muyang He, Jianjun Jiang, Daifeng Cheng

**Affiliations:** 1 Department of Entomology, South China Agricultural University, Guangzhou, 510640 China; 2 Guangxi Academy of Agricultural Sciences, Nanning, China

**Keywords:** *Bactrocera dorsalis*, Symbiont, Plant pathogen, Beneficial microbes

## Abstract

Plant pathogens can build relationships with insect hosts to complete their life cycles, and they often modify the behavior and development of hosts to improve their own fitness. In order to unravel whether some bacteria that can make fruit rot could have developed symbiotic interactions with *Bactrocera dorsalis*, we studied the symbiont bacteria profiles of the fly. We identified the bacterium *Gluconobacter cerinus* strain CDF1 from the ovaries and eggs of the oriental fruit fly *B. dorsalis* and the amount of *Gluconobacter cerinus* strain CDF1 increased significantly as the ovaries developed and in fruits on which non-sterile eggs were laid. *Gluconobacter cerinus* strain CDF1 addition to bananas fastens the rotting process and its addition to the eggs fastens their development/hatching rate. All in all, our data suggest that *Gluconobacter cerinus* strain CDF1 is beneficial to the fruit fly.

## Introduction

Insects and plants have co-existed for more than 400 million years (Sugio et al. 2014), and plant–insect interactions are considered to be one of the most primitive and co-evolved systems (Bronstein et al. 2006). The interaction between plants and insect herbivores is viewed as an arms race: While insects must face plant defenses and evolve strategies to overcome them, plants tend to reduce herbivores by diverse mechanisms (Bronstein et al. 2006; Sugio et al. 2014). Plants can influence the behavior, growth and reproduction of herbivorous insects by changing nutrients and secondary metabolites (Ali and Agrawal 2012; Mithöfer and Boland 2012; Dawkar et al. 2013). In response, insects on plants can then adapt in various ways (Potting et al. 1995; Strauss et al. 2013). For example, studies have shown that the types of toxins produced by plants and the response modes of herbivorous insects can affect adaptive responses, which may result in convergent evolution at the molecular level (Susanne et al. 2012).

Researchers have found that plants and insects establish different types of relationships with microbial associates that influence the outcomes of plant–insect interactions (Sugio et al. 2014). Plant primary and secondary metabolites and/or plant defenses against insects may be modulated by microbes, which may benefit the insects. Similarly, microbes may affect insect biology, including changing metabolism and behavior. For example, *Rickettsia* has been found to spread rapidly (6 years) in natural populations of the sweet potato whitefly *Bemisia tabaci* in the southwestern USA and to considerably increase the performance of infected whiteflies compared with uninfected whiteflies (Himler et al. 2011). Likewise, the Enterobacter *Erwinia* sp., which infects the thrips *Frankliniella occidentalis*, can be beneficial for its hosts depending on which plant the thrips are feeding upon (Vries et al. 2004). Many insect-associated microorganisms can even promote the capacity of insects to utilize diets with low or unbalanced nutritional content by providing specific nutrients that the insect cannot synthesize, including essential amino acids, B vitamins and sterols (Douglas 2015). For example, when *Buchnera* bacteria are eliminated from aphids using antibiotics, the aphids lose the ability to synthesize essential amino acids (Febvay et al. 1999; Douglas et al. 2001). The microorganisms located in the hindgut fermentation chamber of insects mediate the slow enzymatic degradation of the cellulose and hemicellulose components of plant tissue to sugars, which are then available to the insects (Calderóncortés et al. 2012).

The Oriental fruit fly *Bactrocera dorsalis* (Hendel) (Diptera:Tephritidae) is a notorious pest that damages a wide range of fruit and other horticultural products (Hollingsworth et al. 2003; Yee and Goughnour 2008). Damage from *B. dorsalis* can cause fruit to rot and drop, leading to economic losses (Jiang et al. 2011). Many studies have shown that symbiotic bacteria of insects can decompose plant tissues to supplement essential nutrients for their insect hosts (Douglas et al. 2006; Feldhaar 2011; Price et al. 2014). Thus, we hypothesized that some bacteria that cause fruit rot could have developed symbiotic interactions with *B. dorsalis.* However, complementary data on the bacterial communities—data needed to evaluate the hypothesis effectively—were not collected. Here, we concurrently analyzed the bacterial and developmental profiles of *B. dorsalis* and the rot effect in fruits to determine whether they co-varied.

Our results demonstrate that the bacterial communities in *B. dorsalis* are dominated by certain bacteria and that the development of *B. dorsalis* and fruit rot are affected by *Gluconobacter* bacteria, which can cause plant pink disease (Kontaxis 1978; Rohrbach 1989). This study suggests that the *Gluconobacter* bacterium in *B. dorsalis* is sufficient to increase the adaptability of the host. Moreover, it provides a new example of a bacterial plant pathogen that can also act as a beneficial symbiont for insects.

## Materials and methods

### Insects

The *B. dorsalis* used in this study were originally collected in April 2015 from a carambola (*Averrhoa carambola*) orchard in Guangzhou, Guangdong Province and were reared in the laboratory for 1 generation under the following conditions: 25 ± 1 °C, 16:8 h light: dark cycle, 70–80% RH, and a maize-based artificial larval diet containing 150 g of corn flour, 150 g of banana, 0.6 g of sodium benzoate, 30 g of yeast, 30 g of sucrose, 30 g of paper towel, 1.2 mL of hydrochloric acid and 300 mL of water. The adult diet consisted of water, yeast hydrolysate and sugar.

### *Bactrocera dorsalis* adaptability and effect on fruits after eggs were treated with antibiotic

To determine whether the bacteria in *B. dorsalis* affect fly adaptability and fruit rot, 30 newly laid eggs were soaked with streptomycin solution (0.5 mg/mL) for 1 h. In order to show the effect of the antibiotic on the bacteria inside the eggs, bacteria in streptomycin (0.5 mg/mL) treated eggs (5 eggs were grinded and diluted in 1 mL sterile water) were cultivated on the Luria–Bertani culture medium, and the bacteria in normal eggs were cultivated as control. Four replicates were performed. And antibiotic treated eggs were collected, immediately dried with absorbent paper, and inoculated into newly picked fruit (guava and mango). After inoculation, the states of the fruits were recorded every 2 days. Six days after inoculation, the hatching rate and development of the larvae were compared. Three replicates were performed. For controls, three replicates with water-soaked eggs were also performed.

### Bacterial operational taxonomic unit (OTU) surveys

To identify the potential effect of symbiotic bacteria on *B. dorsalis* adaptability and fruit rot, the ovaries of 3 female flies from the same population were dissected and collected every 2 days since emergence. The flies were soaked in 75% alcohol for 3 min to avoid the contamination of the environment. The soaked flies were dissected under a stereo-microscope in 75% alcohol, and the ovaries were washed in alcohol and transferred into centrifuge tubes containing DNA extraction buffer. A portion of the rot guava fruit samples were also collected every 2 days after inoculation with fly eggs. Sample DNA was extracted using a DNA extraction kit (Tiangen, Beijing, China) following the manufacturer’s instructions. Bacterial 16S rRNA genes were amplified from the extracted DNA by PCR, using two broadly conserved, degenerate primers targeting the V3 + V4 variable region of the 16S gene (F:5′-CCTACGGGNGGCWGCAG-3′ and R:5′-GGACTACHVGGGTATCTAAT-3′). Each sample was analyzed in a total reaction volume of 25 μL containing 2.5 μL of Takara 10 × Ex Taq buffer, 1.5 μL of Mg^2+^ (25 mM), 2 μL of dNTPs (2.5 mM), 0.25 μL of Takara Ex Taq (2.5 U/μL), 0.5 μL of each primer (10 µM), 16.75 μL of ddH_2_O and 1 μL of template. The PCR amplifications were performed with a 2-min incubation at 95 °C followed by 30 cycles at 94 °C for 30 s, 57 °C for 30 s, and 72 °C for 30 s, with a final 5-min extension at 72 °C. The PCR products were purified using a QIAGEN MinElute PCR Purification Kit to remove unincorporated primers and nucleotides. An ND-1000 microspectrophotometer (NanoDrop Technologies, Wilmington, DE, USA) was used to measure the concentration of the purified DNA. Adapters were added to the purified DNA to build a library for sequencing using an Illumina sequencing kit and an Illumina MiSeq sequencer (Illumina, San Diego, CA, USA). After sequencing, the data were filtered to remove low-complexity sequences (such as poly(A) sequences) and sequences with Ns. The filtered sequences were termed target sequences (tags). To obtain unique tags and to determine the number of tags in the dataset, the dataset was subjected to redundancy treatment using Mothur software (v. 1.27.0) (Schloss et al. 2009). For taxonomic annotation, an RDP classifier (Huse et al. 2010) was used with naïve Bayes settings; the confidence threshold was set to 0.5. To obtain additional information regarding taxonomic diversity, we subjected the tags to OTU abundance analysis. The number of OTUs was calculated using Mothur software at 97% similarity (Schloss et al. 2009). We first annotated the tags in an OTU. Then the OTU was annotated with taxonomic unit. The taxonomic unit annotation information of the OTUs was used to generate OTU abundance profiles for all the samples. The abundance profiles of the OTUs in different samples were used to perform principal component and clustering analyses to investigate the differences between samples using R software. To ensure accuracy the OTUs with abundance lower than 0.001 in all samples were deleted.

### Difference analysis of samples based on classification level

To identify biomarkers between the different samples, LEfSe (https://huttenhower.sph.harvard.edu/galaxy/) analysis was performed using online software (Segata et al. 2011). For LEfSe analysis, the alpha value for the factorial Kruskal–Wallis test among classes and the alpha value for the pairwise Wilcoxon test between subclasses were set to 0.05. Finally, the threshold of the logarithmic LDA score for discriminative features was set to 4.0.

### Bacterial isolation and culture

Newly laid eggs of *B. dorsalis* were collected and immediately soaked in 70% ethanol for 1 min to remove surface bacteria. Then, the eggs were collected in a sterile centrifuge tube to which 200 μL of sterile water was added. The eggs were ground with sterile grinding pestles, and 20 μL of fluid was coated and cultivated on a culture medium plate (1% glucose, 0.5% ethanol, 1.5% agar, 0.8% yeast extract, and 0.3% acetic acid) for 24 h at 30 °C. Colonies with the same morphology were selected for subculturing. The pure cultures were inoculated into liquid medium (1% glucose, 0.5% ethanol, 0.8% yeast extract, and 0.3% acetic acid), and the liquid cultures were stored in 25% glycerol solution at − 80 °C. And the sensitive of the isolated bacterium to streptomycin (0.5 mg/mL) test was done.

### 16S rRNA amplification and identification

Bacteria were collected from pure cultures for the extraction of genomic DNA using a Bacterial Genome DNA Extraction Kit (Tiangen, Beijing, China) according to the manufacturer’s instructions. The 16S rRNA amplification was performed in a total reaction volume of 50 μL with 0.4 μL of DNA polymerase (5 U/μL), 5 μL of 10 × PCR buffer, 4 μL of dNTPs (2.5 mM), 1 μL of each primer (10 µM) (F: 5′-AGAGTTTCATCCTGGCTCAG-3′ and R: 5′-TACGGTTAXXTTGTTACGACTT-3′), 3 μL of DNA template and 33.6 μL of ddH_2_O. The PCR amplification was performed using an Eppendorf thermal cycler and began with a 5-min incubation at 95 °C followed by 35 cycles of 95 °C for 1 min, 55 °C for 1 min and 72 °C for 2 min, with a final extension at 72 °C for 10 min. The PCR products were confirmed by electrophoresis in 0.8% agarose gel and purified with a Gel DNA Mini Purification Kit (Tiangen, Beijing, China). The purified DNA was ligated into the T vector (Tiangen, Beijing, China) and transformed into Top10 *E. coli* cells (Tiangen, Beijing, China) according to the manufacturer’s instructions. The transformed cells were spread on LB agar plates and, after antibiotic selection and blue/white staining, colonies were selected for colony PCR and direct sequencing. The sequences were subjected to a BLAST search against the NCBI database for sequence-homology analysis.

### Effect of *Gluconobacter cerinus* strain CDF1 on fruit

To test the effect of Gluconobacter cerinus strain CDF1 (Accession Number: KX578017 in Genbank, GDMCC 1.1207) which is now deposited in the publicly accessible culture collection GDMCC (Guangdong Culture Collection Centre of Microbiology, China) on fruit, 10 µL of *Gluconobacter cerinus* strain CDF1 inoculum was coated on a banana. The fruits were incubated for 3 days at 30 °C, and banana rot was recorded. As a control, bananas coated with sterile fluid medium were also prepared and incubated for 3 days at 30 °C.

### Fly adaptability and fruit rot rate after eggs were inoculated with *Gluconobacter cerinus* strain CDF1

The *Gluconobacter cerinus* strain CDF1 inoculum was prepared by selecting and incubating a colony of *Gluconobacter cerinus* strain CDF1 in culture medium (1% glucose, 1.5% agar, 0.8% yeast extract) at 30 °C. Next, newly laid fly eggs were collected and soaked in *Gluconobacter cerinus* strain CDF1 inoculum for 1 h. Subsequently, the eggs were placed into the artificial diet; each treatment received 50 eggs. After 5 days, the number and weight of the fly larvae were recorded (5 replicates). As a control, newly laid fly eggs soaked in pure water were also prepared and placed into the artificial diet.

### Statistical analysis

The differences between treatments and controls were compared by independent samples t-tests or one-way analysis of variance (ANOVA) followed by Tukey’s test for multiple comparisons. The differences were considered significant at P < 0.05. The data were analyzed using SPSS software. To identify biomarkers between different samples, LEfSe (Linear discriminant analysis Effect Size) (https://huttenhower.sph.harvard.edu/galaxy/) analysis was performed using online software. For LEfSe analysis, the alpha value for the factorial Kruskal–Wallis test among classes and the alpha value for the pairwise Wilcoxon test between subclasses were set to 0.05.

## Results

### The bacterial profiles of fly ovaries

**Fig. 1:**
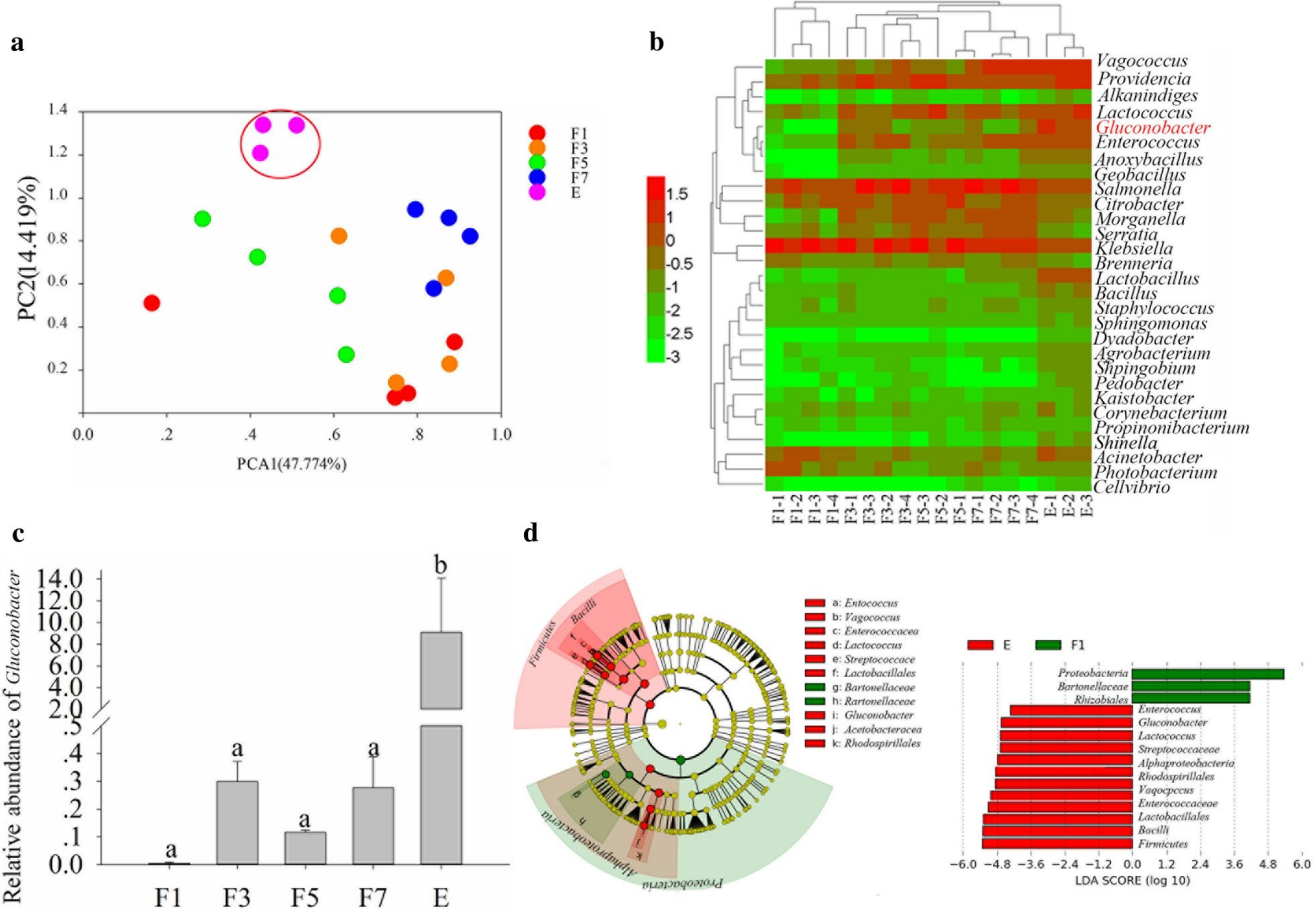
Bacterial communities in the ovaries of flies. **a** Principal components analysis of bacterial communities. **b** Cluster analysis cladogram showing the identified bacteria. The data were log transformed before plotting. **c** Difference in abundance of *Gluconobacter* sp. **d** Key factor screening for differences between samples by LEfSe analysis, the biomarkers with LDA scores above 4.0 were showed in green or red dots in the cladogram. Means (± SEM) that are labeled with different letters are significantly different; F1: ovary of a female fly 1 day after emergence; F3: ovary of a female fly 3 days after emergence; F5: ovary of a female fly 5 days after emergence; F7: ovary of a female fly 7 days after emergence; E: ovary of a female fly with developed eggs

To identify how the flies obtain bacteria that are beneficial to their development and can cause fruit rot, bacterial profiles of fly ovaries at different development stages were investigated. The 16S rRNA gene surveys, using an OTU definition of 97% homologous nucleotide base similarity, revealed that the bacterial communities in fly ovaries were markedly different at different developmental stages. Principal component analysis of bacterial communities also revealed that the bacterial communities in fly ovaries at different developmental stages were markedly different (Fig. 1a). For species annotation, the abundance of *Gluconobacter* increased significantly as ovaries matured (ANOVA: *F* = 4.251, *df* = 17, *P* = 0.02; Fig. 1b, c). Moreover, the results of LEfSe analyses indicated that *Gluconobacter* increased significantly as the ovary getting mature and was the key factor differentiating the mature ovaries (Fig. 1d), which may indicate that *Gluconobacter* is a key factor affecting fly development. Moreover, the higher taxonomic units of *Gluconobacter* were identified as the biomarkers in the mature ovaries due to the increased amount of *Gluconobacter*.

### The bacterial profiles of fruit inoculated with fly eggs

**Fig. 2:**
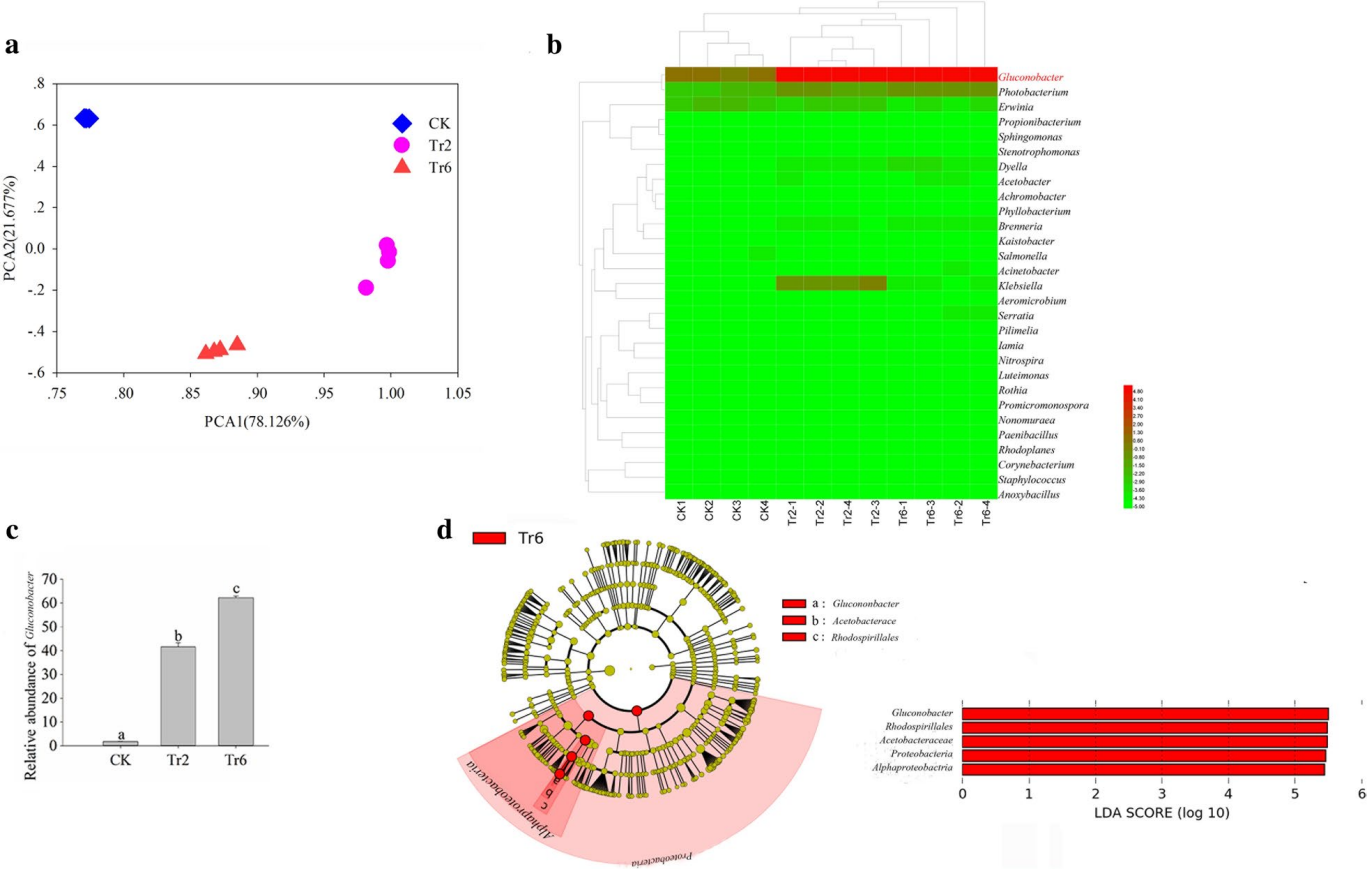
Bacterial communities in guava inoculated with fly eggs. **a** Principal components analysis of bacterial communities. **b** Cluster analysis cladogram showing the identified bacteria. The data were log transformed before plotting. **c** Difference in abundance of *Gluconobacter* sp. **d** Key factor screening for differences between samples by LEfSe analysis, the biomarkers with LDA scores above 4.0 were showed in green or red dots in the cladogram. Means (± SEM) labeled with different letters are significantly different; CK: guava fruit sample collected 1 day after inoculation with fly eggs; Tr2: guava fruit sample collected 2 days after inoculation with fly eggs; Tr6: guava fruit sample collected 6 days after inoculation with fly eggs

To further identify the potential function of *Gluconobacter*, the bacterial profiles of fruit inoculated with fly eggs were studied using 16S rRNA sequencing. We found that the profiles of bacterial communities in guava fruit inoculated with fly eggs were significantly different as the larvae hatched and fed on the fruit (Fig. 2a). When the OTUs were annotated with genus information, abundance profiles revealed that *Gluconobacter* exhibited the greatest difference when larvae were feeding in the guava; abundance of the bacterium was larger as the flies developed and fruit began to rot (ANOVA: *F* = 777.392, *df* = 11, *P* < 0.001; Fig. 2b, c). Moreover, LEfSe analyses indicated that the amount of *Gluconobacter* increased significantly as the fruit get rotting was the key factor differentiating guava fruit fed on by larvae for different durations (Fig. 2d) and the higher taxonomic units of *Gluconobacter* were also identified as the biomarkers in the rot fruits due to the increased amount of *Gluconobacter*. These results may indicate that the *Gluconobacter* bacterial communities can cause rot in guava and were beneficial for fly development.

### *Gluconobacter* sp. isolation and identification

**Fig. 3:**
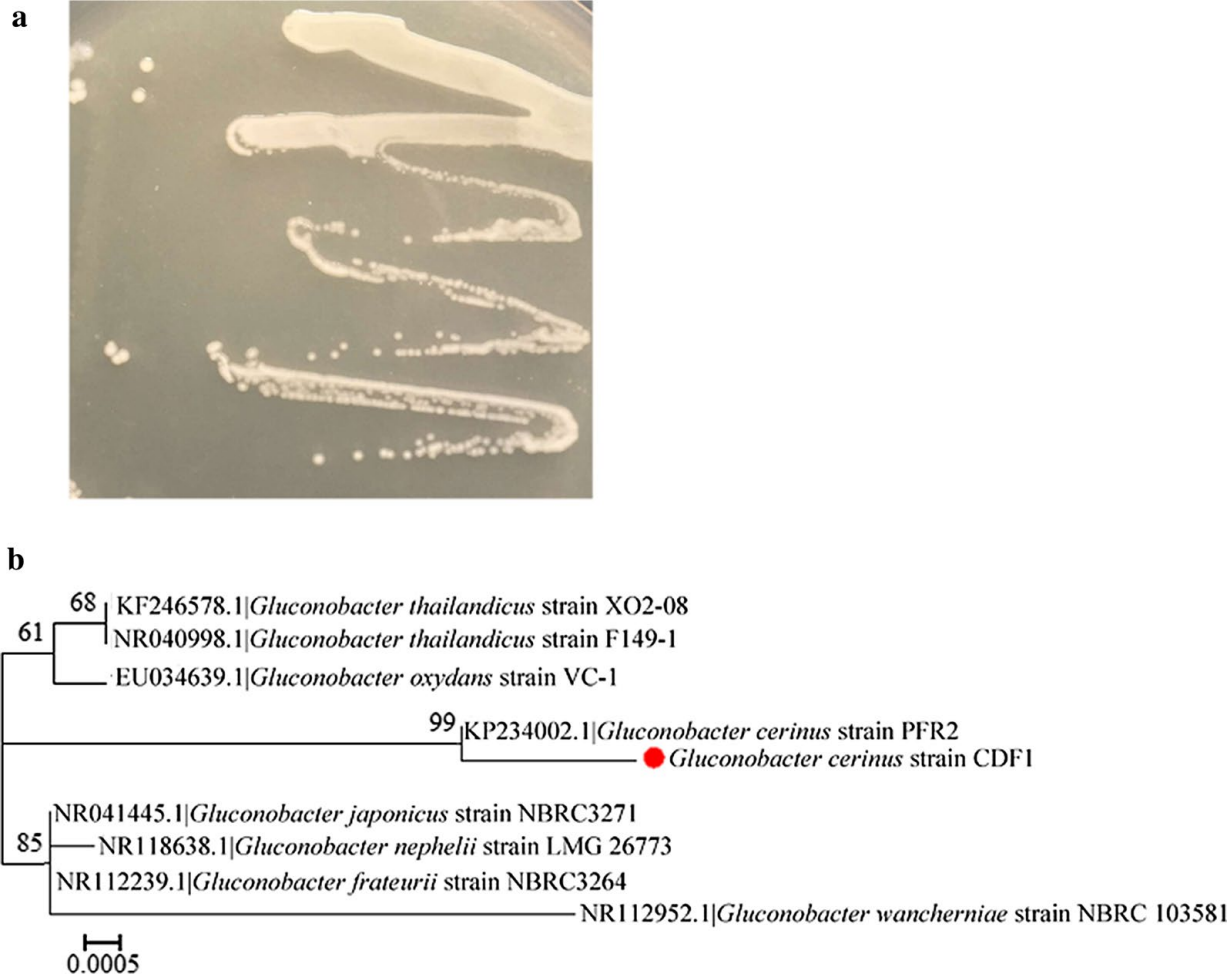
Gluconobacter sp. isolation and identification. **a** Colony characteristics on agar plates for a bacterium isolated from the ovaries and eggs of flies. **b** Phylogenetic relationships of the symbiotic *Gluconobacter cerinus* strain CDF1. The red spot indicates the *Gluconobacter cerinus* strain CDF1. A maximum likelihood phylogeny inferred from 1377 aligned nucleotide sites in 16S rDNA gene sequences is presented with bootstrap values

To reveal the function of *Gluconobacter* sp., subculturing was performed on specific agar flat plates. Then, bacteria were identified by 16S rRNA sequencing. After 48 h, *Gluconobacter* sp. colonies grown from fly egg isolates were identified by their white color, raised center, clean margin, and smooth, wet surface (Fig. 3a). Subsequent 16S rRNA amplification and sequencing yielded a fragment of 1377 bp (Genbank Accession Number: KX578017). Based on a BLAST search against GenBank, the 16S rRNA sequence exhibited a 99% identity match with the *Gluconobacter cerinus* strain PFR2; we named the strain *Gluconobacter cerinus* strain CDF1 (Fig. 3b).

### Antibiotics change the adaptability of fruit flies and the rot rate of fruit

**Fig. 4:**
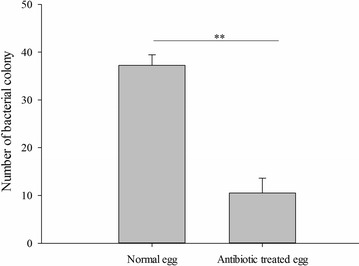
Number of cultivated *Gluconobacter cerinus* strain CDF1 colonies separated from the eggs treated with antibiotic. **p < 0.01

**Fig. 5:**
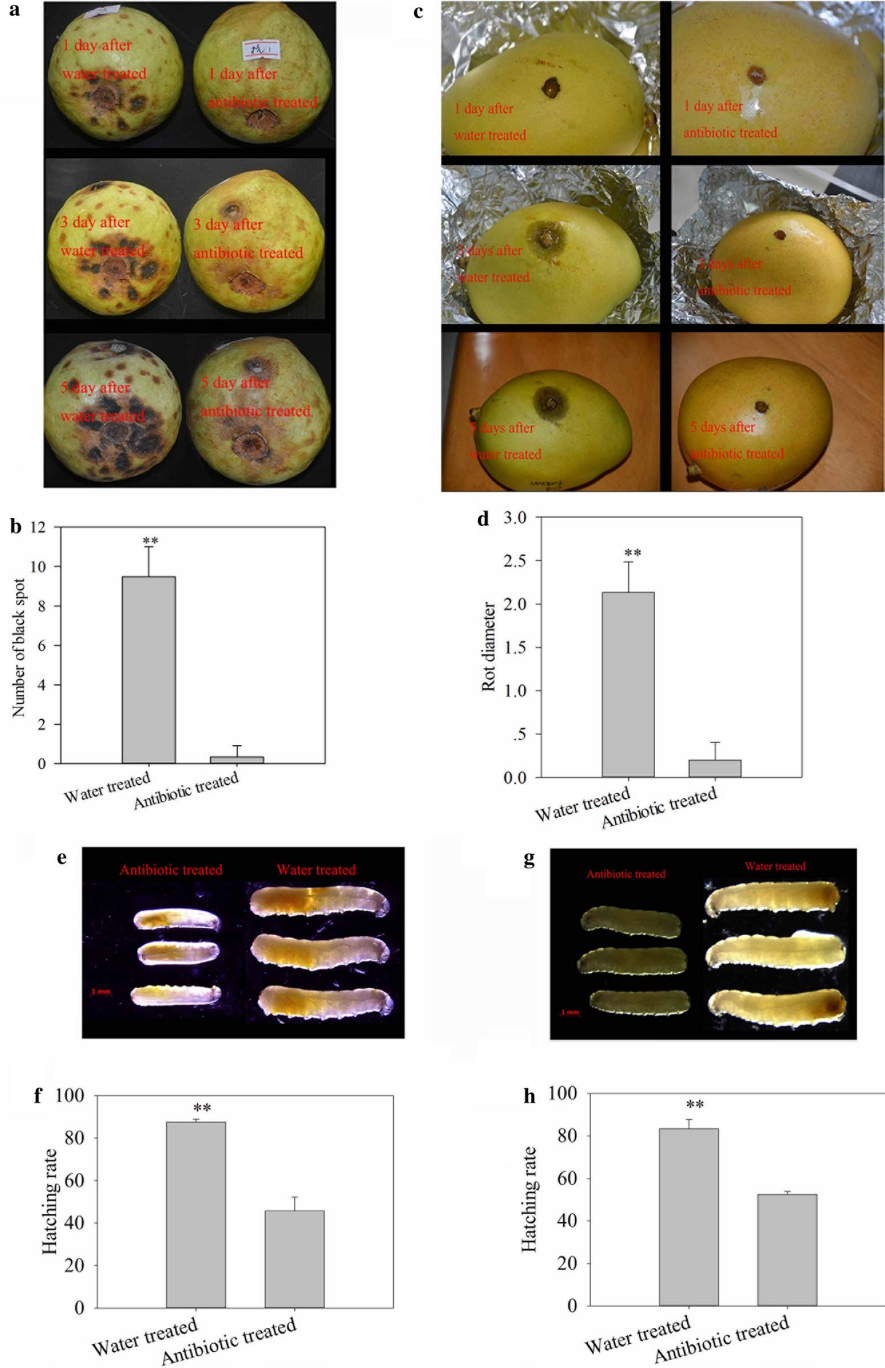
Effects on fruit, larvae development and hatching after fly eggs were treated with antibiotic. **a** Effects on guava after inoculating the antibiotic-treated eggs. **b** Number of black spot on guava inoculated with eggs. **c** Effects on mango after inoculating the antibiotic-treated eggs. **d** Rot diameter of mango inoculated with eggs. **e** Body length of larvae hatching in guava. **f** Hatching rate of eggs in guava. **g** Body length of larvae hatching in mango. **h** Hatching rate of the eggs in mango. **p < 0.01

To manipulate symbiont infection status, *B. dorsalis* eggs were surface-sterilized by soaking in streptomycin solution. The result showed that the antibiotic had significantly decreased the number of *Gluconobacter cerinus* strain CDF1 colonies cultivated on the LB culture medium (independent samples *t* test, *t* = 14.01, *df* = 6, *P* < 0.001, Fig. 4). And the procedure consistently resulted in slower guava and mango fruit rot (Fig. 5a, c), more black spot on guava and larger rot area on mango were recorded on the fruit without antibiotic treatment (in guava: independent samples t-test, *t* = 9.899, *df* = 4, *P* = 0.001; in mango: independent samples t-test, *t* = 8.286, *df* = 4, *P* = 0.001, Fig. 5b, d). Moreover, the development of *B. dorsalis* was significantly affected; in guava and mango fruit, antibiotic treatment resulted in slower larval development (Fig. 5e, g) and hatching rate (in guava: independent samples t-test, *t* = 6.25, *df* = 4, *P* = 0.003, Fig. 5f; in mango: independent samples t-test, *t* = 6.645, *df* = 4, *P* = 0.003, Fig. 5h). These results may indicate that symbiotic bacteria in eggs are important to fly development and intensify fruit rot.

### *Gluconobacter* sp. strain CDF1 fastens fruit rotting and fruit fly development

**Fig. 6:**
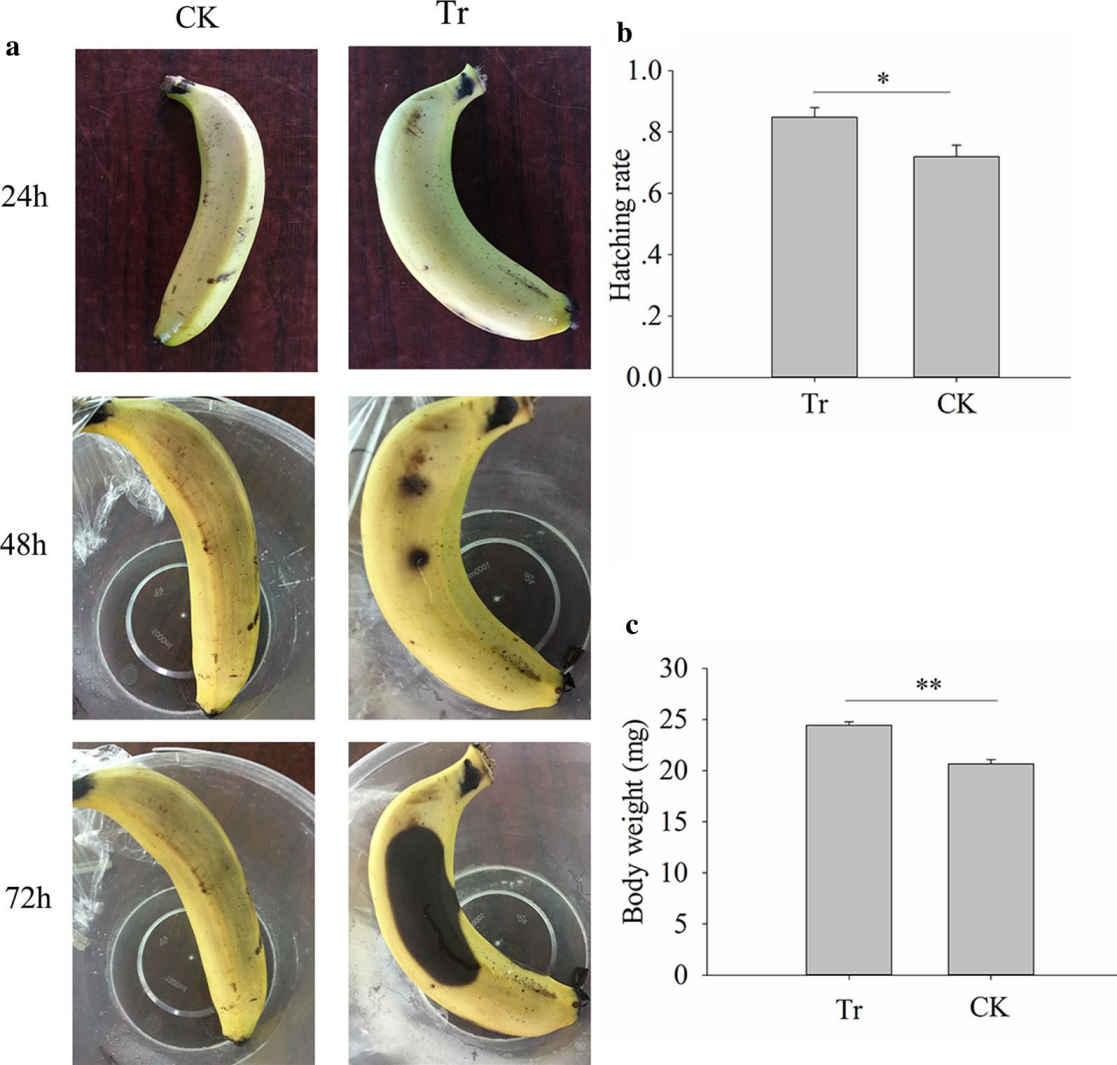
Effects of *Gluconobacter cerinus* strain CDF1 on bananas and fly development. **a** Effect of *Gluconobacter* sp. on banana. **b** Effect of *Gluconobacter cerinus* strain CDF1 on fly hatching rate. **c** Effect of *Gluconobacter cerinus* strain CDF1 on larval body weight. Tr: banana or fly eggs painted with *Gluconobacter cerinus* strain CDF1; CK: banana or fly eggs painted with sterile water. *p < 0.05; **p < 0.01

Adding *Gluconobacter cerinus* strain CDF1 inoculum to bananas caused rot to occur quickly (Fig. 6a). Moreover, the hatching rate and the development of the flies were enhanced by soaking fly eggs in *Gluconobacter cerinus* strain CDF1 inoculum (independent sample t test, *t* = 2.622, *df* = 8, *P* = 0.031, Fig. 6b; *t* = 6.916, *df* = 38, *P* < 0.001, Fig. 6c). These results verified the function of *Gluconobacter* sp. strain CDF1 in enhancing fly development and fruit rot.

## Discussion

Our study tested a typical host-symbiont association between the plant pathogen *Gluconobacter cerinus* strain CDF1 and its host, *B. dorsalis*. We demonstrated that (i) antibiotic treatment inhibited the fly hatching rate and growth of flies and the rot rate of fruit, and (ii) *Gluconobacter* bacteria can affect the hatching rate and growth of flies and the rot rate of fruit. Our results demonstrated a mechanism by which a plant pathogen and an insect host experience a beneficial symbiotic relationship.

Unlike mammals, insects such as *Drosophila* harbor no more than 30 sequence-based differentiated taxa or OTUs on average (Chandler et al. 2011; Colman et al. 2012; Jones et al. 2013). Proteobacteria and, in particular, Enterobacteriaceae have been identified as the most prevalent microbial associates of many insects (Moran et al. 2008). Moreover, *Drosophila*, a related dipteran, is similarly dominated by an acetic acid bacterium (Chandler et al. 2011). The identified *Gluconobacter cerinus* strain CDF1 belongs to *Acetobacteraceae* in our study supports the conclusion in *Drosophila*. Bacterial communities located in different parts of the host have different functions. Many bacteria harbored in the insect gut play important nutritional roles to affect the development of the insects, either directly or indirectly (Dillon and Dillon 2004), while other bacteria that infect insect reproductive organs can alter the host survive in many ways (Engelstädter and Hurst 2009). Thus the function of *Gluconobacter cerinus* strain CDF1 in *B. dorsalis* supports this conclusion.

Insects have established different types of relationships with microbial associates that influence the outcomes of insect interactions (Sugio et al. 2014). Microbes can change the biology of their insect hosts, including both host metabolism and behavior. *Rickettsia*, an insect symbiont, manipulates insect host reproduction, considerably increasing the performance of infected whiteflies compared with uninfected whiteflies (Himler et al. 2011). Bacteria can provide increased protein consumed as food for insects, higher levels of rot may make feeding easier with less host enzymes needed to break down cells, or allows larvae to spread out more and not have as much competition with other larvae, or sugar in fruit may be somewhat toxic and bacteria consume sugar easing effect on larvae (Huang and Douglas 2015). And in our research, we found that *Gluconobacter cerinus* strain CDF1, a plant pathogen, increased the fitness of fruit flies and had negative effects on fruit, thus we need more evidence to unravel the mechanism under this phenomenon.

Microbes can manipulate plants to attract insect vectors (Jordano 2012). By fermenting or damaged fruit esters, alcohols, acids or carbon dioxide can be produced, these fermenting substrates are not only used as food resource but also as mating site and thus attract the *Drosophila* (Hamby and Becher 2016). In the beetle, study revealed that microbial communities that are enriched with genes involved in terpenoids (synthesized by pine and are toxic to beetles) degradation are strongly associated *Dendroctonus ponderosae* (Adams et al. 2013). And the bacteria associated with *D. ponderosae* were shown to metabolize monoterpenes and diterpene acids (Boone et al. 2013). Some microbes can not only suppress plant defense systems but also change plant architecture and/or physiology to lure insects (Sugio et al. 2014). With a series of complex action mechanisms, aster yellows phytoplasma can change the physiology of its host plant, making it more attractive for oviposition by the leafhopper vector *M. quadrilineatus* (Bai et al. 2009; Sugio et al. 2011; Maclean et al. 2014). Plant volatile compositions can even be changed by some microbes and thus attract insect. The apple volatile composition is changed by a phytoplasma strain, *Ca. P.* mali, to lure the *Cacopsylla picta* (Mayer et al. 2008), and infection by Liberibacter can induce the release of methyl salicylate to attract *D. citri* (Mann et al. 2012). However, the mechanisms that microbes change the plant volatiles have not been fully reported. Thus, more evidence is needed to prove whether *Gluconobacter cerinus* strain CDF1 can change the physiology or the volatiles of fruits to attract flies.

## Abbreviations

OTU: operational taxonomic unit
ANOVA: one-way analysis of variance
LEfSe: linear discriminant analysis effect size
